# Polyadenylation landscape of in vivo long-term potentiation in the rat brain

**DOI:** 10.1261/rna.080485.125

**Published:** 2025-12

**Authors:** Natalia Gumińska, Francois P. Pauzin, Bożena Kuźniewska, Jacek Miłek, Patrycja Wardaszka-Pianka, Paweł S. Krawczyk, Seweryn Mroczek, Sebastian Jeleń, Patrick U. Pagenhart, Clive R. Bramham, Andrzej Dziembowski, Magdalena Dziembowska

**Affiliations:** 1 Laboratory of RNA Biology, International Institute of Molecular and Cell Biology, Warsaw 02-109, Poland; 2 Department of Biomedicine, University of Bergen, Bergen NO-5020, Norway; 3 Mohn Research Center for the Brain, University of Bergen, Bergen NO-5020, Norway; 4 Faculty of Biology, University of Warsaw, Warsaw 02-096, Poland; 5 Centre of New Technologies, University of Warsaw, Warsaw 02-097, Poland

**Keywords:** long-term potentiation, polyadenylation, hippocampus, Oxford Nanopore sequencing, nonadenosine

## Abstract

Local protein synthesis in neurons is vital for synaptic maintenance and plasticity, yet the regulatory mechanisms, particularly cytoplasmic polyadenylation, are not fully understood. This study used nanopore sequencing to examine transcriptomic responses and 3′-end dynamics in rat hippocampal long-term potentiation (LTP) in vivo and in synaptoneurosomes after in vitro stimulation. Our long-read transcriptomic data set allows for detailed analysis of mRNA 3′-ends, poly(A) tail lengths, and nucleotide composition. We observed dynamic shifts in polyadenylation site preference post-LTP induction, with significant poly(A) tail lengthening restricted to transcriptionally induced mRNAs. The poly(A) tails of these genes showed increased nonadenosine abundance. In synaptoneurosomes, chemical stimulation led to the shortening of poly(A) tails on preexisting mRNAs, indicating translation-induced deadenylation. This also includes transcripts, which were previously reported to undergo stimulation-induced cytoplasmic polyadenylation, like *Camk2a*. Additionally, we discovered a group of neuronal transcripts with poly(A) tails abundant in nonadenosine residues. These tails are semi-templated and derived from extremely adenosine-rich 3′UTRs. This study provides a comprehensive overview of mRNA 3′-end dynamics during LTP, offering insights into post-transcriptional regulation following synaptic activation of plasticity in neurons.

## INTRODUCTION

Synaptic plasticity, the activity-dependent modification of synaptic efficacy, is critical for learning and memory processes (Citri and Malenka 2008). In the mammalian brain, long-term potentiation (LTP) and long-term depression (LTD) are canonical models for strengthening and weakening of synaptic transmission, respectively. LTP of excitatory synapses in the hippocampus is divided into early and late phases, distinguished by their duration and underlying mechanisms. After NMDA receptor-dependent LTP induction, expression of early phase LTP depends on post-translational modification and trafficking of preexisting proteins, such as AMPA receptors membrane shuttling, and does not require massive transcriptome remodeling with only selected immediate early genes being induced. Late-phase LTP, on the other hand, involves complex transcriptomic responses and de novo protein synthesis, which enables stable structural and functional remodeling of synapses.

Pre-mRNA polyadenylation is a 3′-end processing mechanism for the export of RNAs from the nucleus into the cytoplasm. It contributes to mRNA stability and thus acts as a master regulator of gene expression in eukaryotes (Colgan and Manley 1997). The polyadenosine tail, synthesized by the canonical poly(A) polymerase at the poly(A) site (PAS), is defined by upstream and downstream *cis*-regulatory elements recognized by the cleavage and polyadenylation specificity factor (Di Giammartino et al. 2011; Elkon et al. 2013; Fontes et al. 2017). Most mammalian genes contain multiple PASs, leading to multiple mRNA isoforms (Hoque et al. 2013). In neurons, local translation of mRNAs in dendrites and axons enables the rapid delivery of proteins involved in the regulation of synaptic maintenance, homeostasis, and plasticity (Bramham and Wells 2007). The interplay between alternative polyadenylation sites (APA) defining 3′UTRs, poly(A) tail length, translation, and mRNA decay is fundamental in gene expression regulation. Indeed, translational efficiency, as well as mRNA half-lives, has been shown to vary more than 1000-fold between individual transcripts, demonstrating the range and specificity of the regulation of mRNA stability and abundance (Eisen et al. 2020). Several years ago, it was postulated that the translation of a specific group of mRNAs is activated by cytoplasmic polyadenylation (Du and Richter 2005; Mansur et al. 2021). These transcripts contain cytoplasmic polyadenylation elements (CPEs)—sequence motifs recognized by CPE-binding (CPEB) proteins. CPEBs were shown to initially induce deadenylation, in order to store mRNAs in a dormant state with short poly(A) tails. Upon stimulation, poly(A) tails were shown to be extended, thereby activating translation. In addition to polyadenylation, several other post-transcriptional regulatory mechanisms can regulate local protein synthesis including microRNAs or pathways that influence the activity of translation factors (Bramham et al. 2016; Jasińska et al. 2016; Thomas et al. 2018; Eisen et al. 2022; Cagnetta et al. 2023).

Despite the long-standing interest in poly(A) tails and the regulation of protein synthesis, methods for reliable, genome-wide estimation of poly(A) tail length have only recently been developed (Brouze et al. 2023). Initially, most studies relied on Illumina-based protocols, such as TAIL-Seq and PAL-Seq (Chang et al. 2014; Subtelny et al. 2014). However, DNA polymerase is unable to properly bind to long homopolymer tracts (repetitive sequences) during PCR amplification, resulting in truncated estimates of poly(A) tail length. In addition, these methods require RNA fragmentation, as the sequencer can only read 200–300 bp at a time (Brouze et al. 2023).

Third-generation PacBio sequencing protocols, such as FLAM-Seq and PAIso-Seq, eliminate the upper limits of poly(A) tail length detection and provide a complete overview of the entire mRNA molecule (Legnini et al. 2019; Liu et al. 2019). However, they are still affected by amplification bias from library preparation, which calls into question the reliability of the results. Oxford Nanopore Technologies (ONTs) offers a solution with its direct RNA sequencing (DRS) protocol. Unlike previous methods of poly(A) tail length measurement, DRS does not require amplification, allowing for real-time sequencing of single RNA molecules and generating full-length, strand-specific reads (Krause et al. 2019; Workman et al. 2019). This approach provides highly accurate genome-wide poly(A) tail length estimations, as previously validated by low-throughput methods (Tudek et al. 2021; Brouze et al. 2024; Czarnocka-Cieciura et al. 2024). Moreover, our recently developed algorithm Ninetails enables the analysis of nucleotide composition (Czarnocka-Cieciura et al. 2025; Gumińska et al. 2025). DRS can be complemented by ONT cDNA sequencing, providing higher throughput and requiring less input material, albeit with PCR-introduced biases similar to PacBio. Consequently, the long reads from cDNA sequencing facilitate the study of mRNA regulation, including PAS usage (Pardo-Palacios et al. 2023).

In this study, we used nanopore DRS and cDNA sequencing to examine the dynamics of mRNA poly(A) tail length, alternative polyadenylation, and nucleotide composition of poly(A) tails in response to in vivo LTP induction in the rat dentate gyrus and following in vitro stimulation of isolated synaptoneurosomes. By integrating these methods, we aimed to explore the relative contributions of transcriptional induction, alternative polyadenylation, and cytoplasmic polyadenylation in regulating mRNA 3′-end dynamics. Our data do not support cytoplasmic polyadenylation as a significant contributor to translation regulation, as we were unable to demonstrate poly(A) tail extension in synaptoneurosomes or in vivo poly(A) tail elongation, with the exception of transcriptionally induced genes 10 min post-LTP. In contrast, preexisting mRNAs rather undergo a translation-dependent deadenylation similar to that observed in the majority of somatic cells. Concurrently, we identified a new class of mRNAs with composite poly(A) tails, partially derived from extremely adenosine-rich 3′UTRs. We also provided a valuable resource with newly identified neuronal mRNA isoforms, some of which feature LTP-induced changes in APA site usage.

## RESULTS

### Nanopore sequencing reveals expected changes in gene expression following LTP induction

**FIGURE 1.**
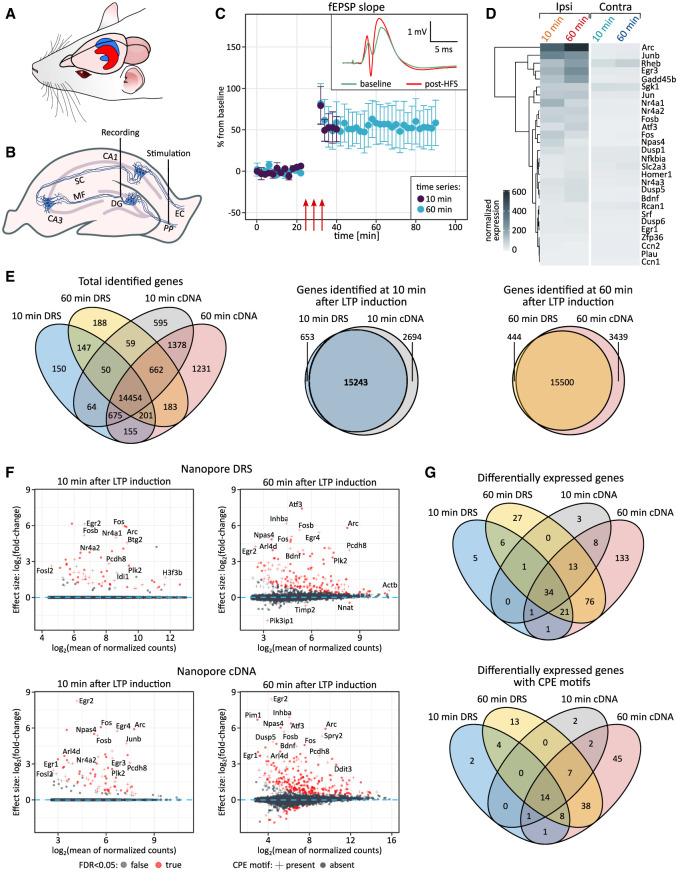
Dynamics of mRNA expression following in vivo hippocampal LTP induction. (*A*) Schematic view of rat hippocampus. LTP was induced in the dentate gyrus of the left hippocampus (ipsilateral, red), while the right hippocampus (contralateral, blue) served as control. (*B*) Schematic view showing placement of electrodes for in vivo stimulation of the medial perforant path fibers from the entorhinal cortex and recording of monosynaptically evoked fEPSPs in the dentate gyrus. (DG) – dentate gyrus, (MF) Mossy fibers, (SC) Schaffer collateral, (PP) perforant path, (EC) entorhinal cortex. (*C*) Representative HFS-induced LTP recordings in anesthetized rats. Time course of fEPSP slope changes (10 and 60 min post-HFS) relative to baseline (mean ± SEM). Red arrows indicate HFS sessions. *Inset*: Traces before (baseline, green) and after HFS (post-HFS, red). (*D*) Expression of IEGs in the dentate gyrus following LTP (Ipsi) versus control (Contra). Colors indicate normalized log_2_ expression levels. (*E*) Overlap of identified genes at 10 and 60 min post-LTP using DRS and cDNA. (*Left*) Venn diagram (all samples). (*Middle* and *right*) Overlap at 10 and 60 min. (*F*) DEGs in the dentate gyrus post-LTP. Log_2_ expression changes at 10 min (*left*) and 60 min (*right*) post-HFS. DEGs (red); genes with CPE motifs (+); genes without CPE motifs (dots). Results from DRS (*top*) and cDNA sequencing (*bottom*). Differential expression was calculated with DESeq2. Log_2_ fold-change estimates were shrunken (*apeglm*) for visualization clarity. (*G*) Overlap of DEGs at 10 and 60 min post-LTP using DRS and cDNA.

To study the activity-induced changes in post-transcriptional regulatory programs in neurons, we used LTP in the rat dentate gyrus (DG) as an established in vivo model for synaptic plasticity. Short bursts of high-frequency stimulation (HFS) were applied to one hemisphere (ipsilateral), while the unstimulated hemisphere (contralateral) served as a control (Fig. 1A). HFS of the medial perforant path input to DG (Fig. 1B) resulted in a rapid and stable potentiation of the field excitatory postsynaptic potential (fEPSP) slope: 57 ± 13% for 10 min and 55 ± 7% for 60 min (Fig. 1C) and a rise in population spike amplitude (Fig. 1C, insert). After in vivo LTP induction (10 and 60 min post-HFS), both ipsi- and contralateral dentate gyri were dissected for RNA extraction. The stimulation paradigm used is known to induce LTP lasting at least 10 h without decrement in anesthetized rats (Panja et al. 2014). Since successful LTP induction leads to the expression of immediate early genes (IEGs) such as *Arc* and *Fos* (Demmer et al. 1993; Guzowski et al. 1999; Maag et al. 2015; Chen et al. 2017; Kim et al. 2018; Patil et al. 2023), we only analyzed the samples with increased expression of these markers in the ipsilateral hemisphere (Supplemental Figs. 1–3A).

To explore the transcriptomic changes induced by neuronal activity after in vivo HFS, we applied two different nanopore sequencing approaches: Nanopore Direct RNA Sequencing (DRS) and Nanopore cDNA from Oxford Nanopore Technologies (ONT). Both methods produce reads encompassing whole molecules of interest, overcoming the limitations of Illumina (Pardo-Palacios et al. 2023). In particular, DRS provides insights into the mRNA inventory without a synthetic proxy, avoiding amplification bias (Workman et al. 2019; Brouze et al. 2023), whereas cDNA sequencing yields higher coverage, ensuring that sufficient data are available for less expressed genes (Sessegolo et al. 2019; Wongsurawat et al. 2022). With DRS, we obtained an average of 0.5 million reads per sample/run, while with cDNA sequencing yielded ∼15 million reads per sample/run (Supplemental Table 1), meeting the analysis requirements. DRS of ipsi- and contralateral dentate gyri confirmed HFS-dependent induction of IEGs, including *Arc* and *Fos,* consistent with qPCR data (Fig. 1D; Supplemental Figs. 1–3A). Based on the literature review, we selected 10 and 60 min post-HFS to capture the early transcription and translation phase critical for the development of late-phase LTP (Otani and Abraham 1989; Messaoudi et al. 2002, 2007; Ryan et al. 2011; Udagawa et al. 2012; Panja et al. 2014; Maag et al. 2015; Patil et al. 2023). The 10 min time point was chosen to capture the onset of the transcriptional responses and enable the assessment of mRNA polyadenylation of preexisting and newly transcribed RNA (Messaoudi et al. 2007; Panja et al. 2014; Patil et al. 2023). We identified 59 and 57 upregulated mRNAs at 10 min post-HFS using DRS and cDNA sequencing, respectively (Fig. 1F, left panels), with no significantly downregulated transcripts. As expected, by 60 min post-HFS, gene expression changes were more pronounced, with 127 and 188 upregulated transcripts detected by DRS and cDNA sequencing, respectively (Fig. 1F, right panels). The overlap between the two methods was substantial: 50% of the differentially expressed genes (DEGs) identified by DRS at 10 min were confirmed by cDNA sequencing, and at 60 min, it was over 80% (Fig. 1E–G; Supplemental Tables 2, 3).

**FIGURE 2.**
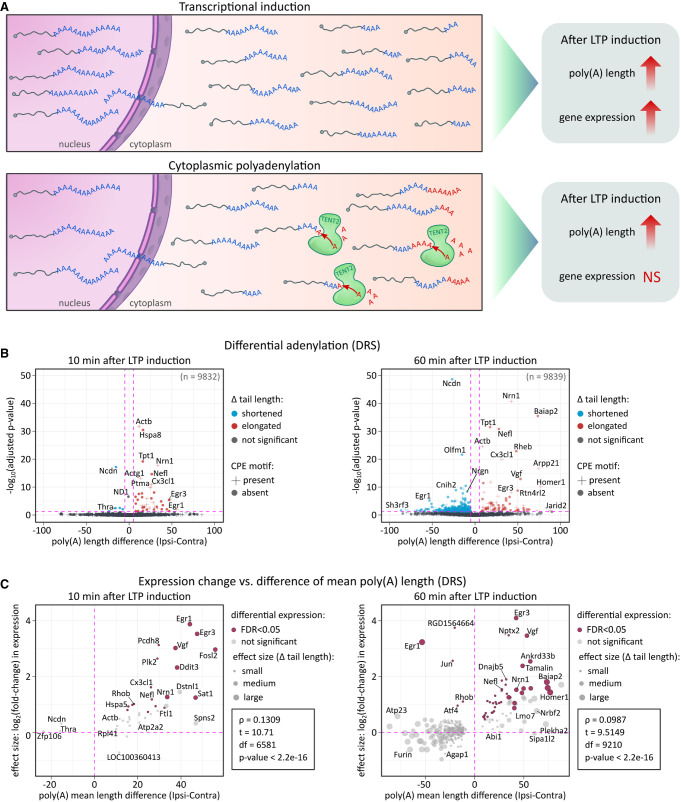
Dynamics of mRNA poly(A) tail lengths following LTP induction. (*A*) Schematic of two potential mechanisms: transcriptional induction and cytoplasmic polyadenylation, underlying observed poly(A) tail length changes. (*B*) Poly(A) tail length distributions at 10 and 60 min post-LTP. *P*-values between conditions were calculated using Wilcoxon signed-rank test, two-sided, α = 0.05, with Benjamini–Hochberg adjustment. The pink dashed lines divide the plot area into sectors based on *P*-value cut-off points and differences in poly(A) tail lengths. (*C*) Differential expression versus differential polyadenylation at 10 and 60 min post-LTP. Genes with significant changes in both are highlighted (maroon). Differential expression calculated using a negative binomial generalized linear model in DESeq2. Only transcripts with statistically significant poly(A) tail length changes are displayed for clarity. Pearson correlation (two-sided) coefficient, *t*-test, degrees of freedom, and *P*-value are provided.

**FIGURE 3.**
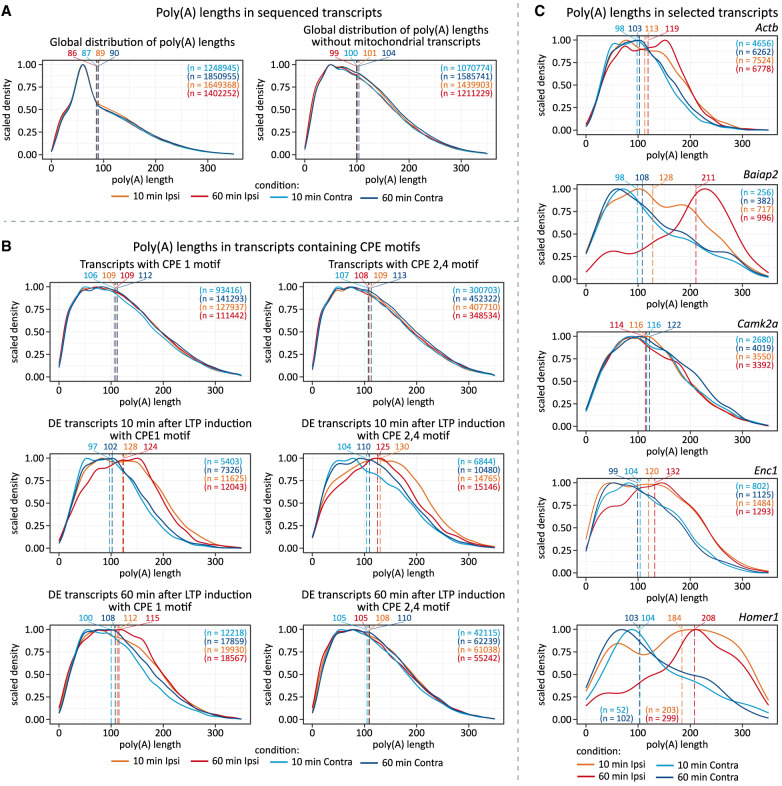
Distribution of poly(A) lengths in selected groups of mRNAs. (*A*) Global distribution of poly(A) lengths: *upper* panel—complete data sets, *bottom* panel—excluding mitochondrially encoded genes. (*B*) Distribution of poly(A) lengths in CPEB targets. (*Upper left*) Transcripts with CPE1 motif (total). (*Upper right*) Transcripts with CPE2,4 motifs (total). (*Middle left*) Transcripts with CPE1 motif differentially expressed 10 min after LTP induction. (*Middle right*) Transcripts with CPE1 motif differentially expressed 60 min after LTP induction. (*Bottom left*) Transcripts with CPE2,4 motifs differentially expressed 10 min after stimulation. (*Bottom right*) Transcripts with CPE2,4 motifs differentially expressed 60 min after LTP induction. All conditions are included in each panel. (*C*) Distribution of poly(A) lengths in selected transcripts associated with neuroplasticity. For *A*–*C*, dashed lines represent median tail lengths for each condition, with median values displayed above the plotting area. The total number of reads per condition (*n*) is also provided.

Numerous specific regulatory motifs reside within mRNA sequences, predominantly in the 3′UTRs. These elements are targets for mRNA-contacting ribonucleoprotein molecules or proteins. Among them, CPEB proteins play important roles in affecting the dynamics of poly(A) tails (de Moor and Richter 1999; McFleder et al. 2017). Therefore, we investigated the presence of a CPE in the DEGs. Indeed, a significant fraction of upregulated transcripts harbor CPE (Fig. 1F,G; see also Figs. 2A, 3B).

We conclude that LTP-inducing stimulation in vivo leads to the expected changes in gene expression as determined by both DRS and cDNA long-read sequencing, providing a basis for a detailed survey of the mRNA 3′-ends and poly(A) tails.

### Dynamics of mRNA poly(A) tail lengths after LTP induction

To investigate poly(A) tail dynamics in activity-dependent synaptic plasticity, we analyzed DRS and cDNA sequencing data sets, considering transcriptional induction and cytoplasmic polyadenylation as potential mechanisms for LTP-induced tail elongation (Fig. 2A). While the former increases both gene expression and polyadenylation, the latter elongates tails post-transcriptionally without affecting mRNA levels.

Indeed, we observed changes in the distribution of poly(A) tail lengths, with a gradual increase in a subset of transcripts affected upon LTP (Fig. 2B). Initially, 10 min post-HFS, a significant change of tail length occurred in 54 genes, of which 44 underwent tail extension. The Cohen's *d* effect size of this elongation was medium for 10 genes (*Nrn1*, *Egr3*, *Sat1*, *Vgf*, *Dstnl1*, *Arpp21*, *Egr1*, *Fosl2*, *Ddit3*, and *Spns2*; with a mean tail length difference between ipsi- and contralateral hemispheres of 42.3 nt), small for 27 (e.g., *Actb*, *Nefl*, *Dnaja1*, *Nefm*, *Nptx1*, *Dclk1I*; with a mean tail length difference between ipsi- and contralateral hemispheres of 20 nt), and negligible for four genes (*Tuba1a*, *Ckb*, *Enc1*, *Hsp90aa1*; with a mean tail length difference between ipsi- and contralateral hemispheres of 7.59 nt). Among them, the most significantly enriched gene ontology (GO) terms were associated with synapse organization, activity, and transport (Supplemental Fig. 3B). In contrast, 60 min post-HFS, a significant change in tail length occurred in 417 genes, 74 of which were elongated. The size of this effect was large for eight genes (*Baiap2*, *Arpp21*, *Homer1*, *Nrbf2*, *Jarid2*, *Plekha2*, *Htra4*, *Mob3a*; with a mean tail length difference between ipsi- and contralateral hemispheres of 74.38 nt), medium for 22 genes (e.g., *Nrn1*, *Rheb*, *Vgf*, *Rtn4rl2*, *Egr3*, *Rbbp7*, *Synpo*, and *Penk*; with a mean tail length difference between ipsi- and contralateral hemispheres of 45.64 nt), small for 40 genes (e.g., *Nefl*, *Dnaja1*, *Nefm*, *Nptx2*, *Dclk1*, *Nrp1*, and *Stmn4*; with a mean tail length difference between ipsi- and contralateral hemispheres of 21.5 nt), and negligible for four genes (*Actb*, *Rpl41*, *Nptx1*, and *Tubb4b*; with a mean tail length difference between ipsi- and contralateral hemispheres of 8.94 nt). The most enriched GO-terms were linked to cell projection and synapse organization, protein trafficking, and neurodevelopment (Supplemental Fig. 3B).

We then compared changes in mRNA expression with changes in average tail length and found a strong correlation. Even at 10 min after LTP induction, poly(A) tail extension is restricted to upregulate genes, arguing against cytoplasmic polyadenylation of preexisting mRNAs (Fig. 2C). Many mRNAs are upregulated at both time points, like (e.g., *Nefl*, *Nrn1*, *Baiap2*, *Homer1*), including known IEGs (e.g., *Egf3*, *Nr4a1*) with accordingly elongated poly(A) tails. Simultaneously, a large number of mRNAs with shortened poly(A) tails at the 60 min time point correlated with their downregulation, suggesting deadenylation-dependent decay. Notably, expression of *Btg2*, a general activator of deadenylation, is among the IEGs induced by the HFS (see Fig. 1F; Mauxion et al. 2008). Despite the general correlation of the poly(A) tail dynamics with the differential expression, we also detected a handful of transcripts in which the elongation of poly(A) tail was independent of upregulation. Those included *Enc1* and *ActB*.

Since mRNAs bound by CPEB proteins are predicted to be the major targets of cytoplasmic poly(A) tail lengthening, we then narrowed our investigation to transcripts containing CPE (recognized by either CPEB1 and/or CPEP2,4) and compared them with the overall changes in poly(A) tail length distribution (Fig. 3A,B; Huang et al. 2023). In general, the presence of CPE motifs did not correlate with poly(A) length distribution. Elongation was only observed on induced DEGs with the strongest effect on potential CPEB1 targets 10 min post-LTP (Fig. 3B; Supplemental Fig. 3C).

Finally, we examined specific transcripts recognized for their significant role in hippocampal synaptic plasticity (Fig. 3C), but did not find clear evidence supporting cytoplasmic polyadenylation as a major contributor to the observed poly(A) dynamics. Notably, this was also the case for *Camk2a* (Ca2^+^/calmodulin-dependent protein kinase 2-α), acknowledged for its activity-dependent polyadenylation (Fig. 3C; Wu et al. 1998; Du and Richter 2005). As *Camk2a* is not transcriptionally induced by LTP, the observed tail length distributions remained largely unchanged between conditions, with median differences falling within the range of expected biological variation. In contrast, the transcriptionally induced *Homer1* and *Enc1* mRNAs exhibited clear tail elongation upon induction (Fig. 3C). This unexpected outcome could potentially be attributed to the relatively lower coverage provided by DRS compared to amplification-based methods. Therefore, we corroborated our findings with our cDNA data. Noteworthy, the DRS and cDNA methods are fully consistent in estimating tail length distributions (Supplemental Fig. 4).

These findings suggest that in our experimental system, the observed poly(A) tail lengthening of certain mRNAs after stimulation is most likely due to polyadenylation of the nascent mRNAs, rather than cytoplasmic extension. Furthermore, none of the mRNAs with elongated tails exhibited a significant reduction in accumulation or a detectable decrease in short-tailed read abundance, which would correspond with previous studies focusing on activation-induced cytoplasmic polyadenylation (Eisen et al. 2022).

### Several mRNAs feature LTP-induced changes in APA site usage

**FIGURE 4.**
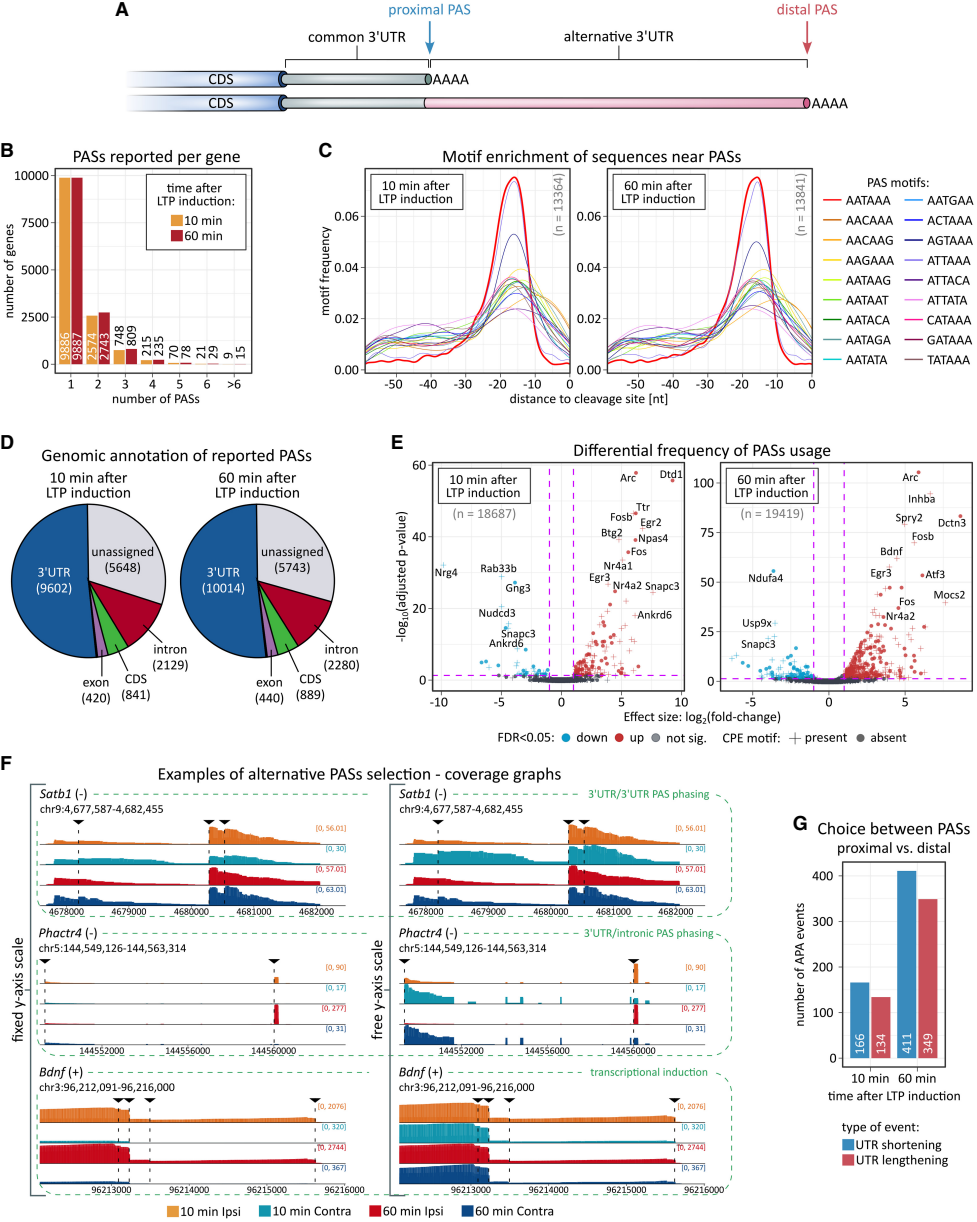
Differential polyadenylation site usage. (*A*) Schematic depiction of 3′UTR containing multiple PASs. (*B*) Number of PASs per gene reported by TAPAS. (*C*) Enriched motifs near detected PASs. (*D*) Genomic location assignment of reported PASs. (*E*) Relationship between PASs usage and expression level. Expression of PASs computed with TAPAS using a negative binomial generalized linear model with the Benjamini–Hochberg adjustment (α = 0.1). (*F*) Examples of genes exploiting multiple PASs with differential frequency due to various mechanisms (*Satb1*—alternative 3′UTRs [lengthening/shortening], *Phactr4*—intronic/3′UTR PASs selection, *Bdnf*—frequency modulated by transcriptional induction). A genome browser-like view of coverages in the 3′UTR region across conditions is shown. (*G*) Usage of distal and proximal PASs in genes with at least two PASs reported. A relative change in expression between conditions for proximal and distal PASs was computed in TAPAS using base-2 logarithms.

Most mammalian genes contain multiple polyadenylation sites (PASs), primarily within the 3′UTR of mRNA (Fig. 4A). Since 3′UTRs often harbor motifs recognized by RNA-binding proteins and miRNA target sites, changes in their length provide an efficient mechanism for regulating gene expression. Alternative polyadenylation (APA) is another post-transcriptional mechanism postulated to be pivotal in LTP (Fontes et al. 2017). Therefore, we investigated whether and how APA site usage changes following in vivo LTP induction in the rat brain (Arora et al. 2021; Ha et al. 2021). To analyze APA profiles, we examined cDNA reads. Given the considerably greater sequencing depth of cDNA sequencing compared to DRS, more precise screening could be achieved. First, to eliminate nanopore-specific artifacts (e.g., fused reads), data sets were curated using pychopper. We then applied TAPAS, a software for detecting APA events in sequencing data (Arefeen et al. 2018).

With TAPAS, we identified nearly 19,000 unique PASs in more than 13,500 genes, of which over 3500 (∼30%) contained at least two PASs, without significant difference in the number of PASs per gene between LTP and control samples (Fig. 4B). Motif enrichment analyses of sequences around PASs confirmed the significant overrepresentation of hexamer motif A[U/A]UAAA and its variants ∼20 nt upstream of PASs (Fig. 4C). Results were comparable for both control and LTP samples 10 and 60 min post-HFS, and they were in agreement with previous studies (Fontes et al. 2017; Wang et al. 2022).

Most of the identified PASs were already known. Over 50% were located in the 3′UTR, ∼18% within transcripts, more than 11% in introns, and ∼3% in exons. Noteworthy, nearly 30% of detected PASs were unassigned to known genomic features, meaning they appear outside annotated 3′UTRs. These likely represent novel isoforms with extended UTRs, consistent with reports demonstrating that UTRs are longer in the nervous system than in other tissues (Fig. 4D; Miura et al. 2013; Tushev et al. 2018). To refine and complement TAPAS predictions, we applied LAPA (Çelik and Mortazavi 2022), generating a high-confidence set of PAS clusters (Supplemental Fig. 5). This provides a comprehensive annotation of 3′UTRs and PASs in the rat brain transcripts, including previously unreported PASs (Supplemental Fig. 5A–E; Supplemental Table 4, PASs coordinates in BED format in Zenodo [Gumińska et al. 2024]).

Next, we analyzed 3′UTR APA events and determined whether the length of 3′UTR was changed globally following LTP induction. With TAPAS, we identified 183 differentially used PASs in 171 genes 10 min after LTP induction and 522 differentially used PASs in 452 genes 60 min post-HFS (Fig. 4E). A core set of 115 genes changing expression between time points included immediate early genes, such as *Arc*, *Fos*, *Jun*, *Btg2*, and *Bdnf*. GO-term analysis revealed that genes with differentially used PASs were enriched in cognition, learning and memory-related processes at 10 min post-HFS, among other terms. At 60 min post-LTP induction, they were associated with rhythmic processes, regulation of phosphorus/phosphate metabolism, and axonogenesis, along with additional enriched terms (Supplemental Fig. 5G).

Multiple regulatory layers control APA, influencing mRNA stability, localization, and translation. For instance, *Satb1* undergoes 3′UTR PAS switching, with the most proximal PAS becoming less favored after stimulation, a pattern most evident after 60 min (Fig. 4F, upper panel). *Phactr4* shifts toward the usage of an intronic PAS upon stimulation, promoting expression of a shorter isoform (Fig. 4F, middle panel). Unlike these, *Bdnf* is an example of mRNA with multiple PASs (Lau et al. 2010), following a transcriptional induction pattern, where the distal PAS isoform is upregulated (Fig. 4F, bottom panel).

We observed a strong link between LTP-induced changes in PAS usage and LTP-induced changes in transcript abundance, with only a minor fraction of PASs altered independently of transcriptional induction (Fig. 4E; Supplemental Fig. 5; compare to Fig. 1F). Furthermore, among all reported APA events, we noticed a general tendency to shorten the 3′UTRs in response to LTP induction, regardless of time point (Fig. 4G), suggesting that proximal PASs are favored, which corresponds with previous findings (Supplemental Table 4; Fontes et al. 2017).

### Neuronal mRNAs with semi-templated poly(A) tails rich in nonadenosine residues

**FIGURE 5.**
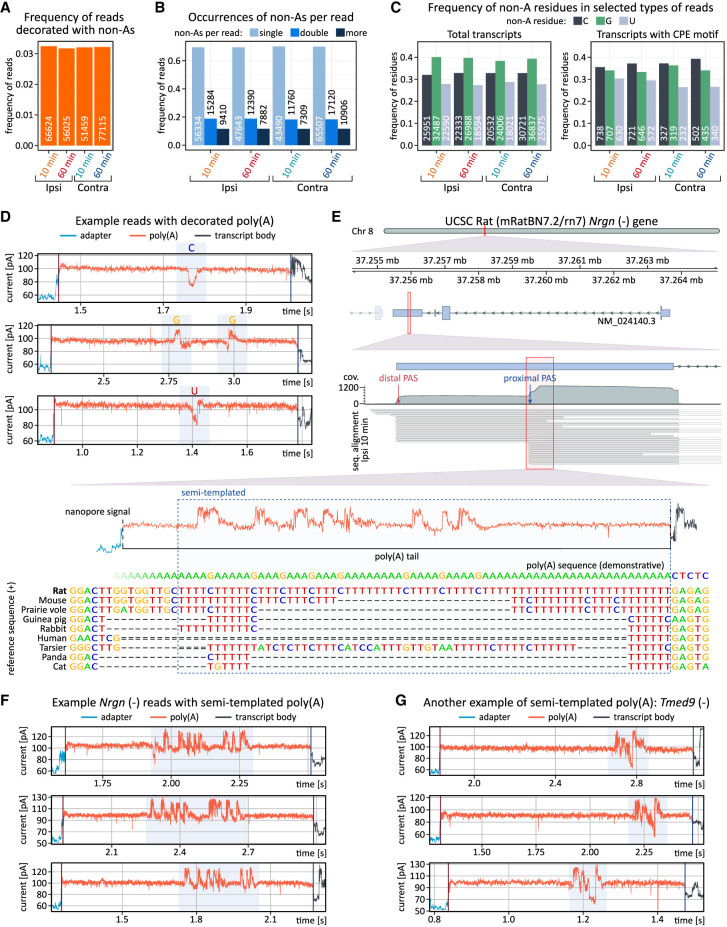
Nonadenosine residues in poly(A) tails. (*A*) Total frequency of reads with poly(A) tails containing nonadenosine residues. (*B*) Frequency of reads with varying numbers of nonadenosine residues per read. (*C*) Frequency of nonadenosine residues (cytidine, guanosine, and uridine) in total data sets and in transcripts with CPE motifs. (*D*) Example nanopore signals with decorated tails. Nonadenosine residues are highlighted. (*E*) Genome browser-like plot of reads aligned to the 3′UTR of the *Nrgn* gene, with nanopore signal indicating semi-templated poly(A) tail origin in an isoform using a proximal PAS and internal nonadenosines (guanosines) in fixed positions. The reference sequence and orthologs from various organisms, as well as the reverse complement mRNA sequence, are shown. The signal is not fully aligned (resquiggled) to the sequence (it cannot be done due to technical limitations of available algorithms). (*F*) Nanopore signals from semi-templated tails of the *Nrgn* isoform with a shorter 3′ UTR, with fixed-position nonadenosine residues (guanosines) highlighted. (*G*) Nanopore signals from semi-templated tails of the *Tmed9* gene, with fixed-position nonadenosine residues (guanosines and cytidines) highlighted. Results presented in all panels were generated using Ninetails.

We then examined the nucleotide composition of poly(A) tails in DRS reads using our recently developed software, Ninetails (Czarnocka-Cieciura et al. 2025; Gumińska et al. 2025). Remarkably, in data from HFS-treated and control tissue, at both 10 and 60 min post-HFS, we observed a fraction of tails containing nonadenosines (Fig. 5A; Supplemental Table 5). These transcripts commonly contained one instance of nonadenosine per tail, and only rarely two or more, coherent with our recent data (Fig. 5B; Legnini et al. 2019; Gumińska et al. 2025). The global proportion of decorated tails was relatively low, slightly above 3%, remaining stable across the conditions (Fig. 5A,B). Subsequently, we focused on certain groups of reads: mitochondrial, DEGs 10 min post-HFS, DEGs 60 min post-HFS, and potential CPEB targets (Supplemental Fig. 6A). In our recent study, we found that mitochondrial transcripts have short but highly decorated tails, reflecting various fidelities of the cellular poly(A) polymerases (Lim et al. 2018; Gumińska et al. 2025). Here, in data from both hemispheres, the proportion of decorated tails in mitochondrial transcripts was also relatively high and nearly equivalent to the total. It also remained stable, as did the distribution of their tail lengths (Fig. 5A, Supplemental Fig. 6A). Conversely, in both DEG subsets and transcripts containing CPE motifs, we observed a higher proportion of decorated reads following LTP induction compared to the corresponding controls (Supplemental Fig. 6A,B). This likely results from the activation of transcriptional mechanisms that drive the synthesis of new molecules with longer tails, where polymerase-induced errors are more frequent (Gumińska et al. 2025).

We also noticed that nonadenosines are more common in longer tails (Supplemental Fig. 6), indicating the possibility of their incorporation by TENT4A/B poly(A) polymerases known for their low fidelity (Supplemental Fig. 6C; Lim et al. 2018; Warkocki et al. 2018; Yu and Kim 2020; Wen et al. 2023). It is supported by the fact that at the global level guanosine is most prevalent, followed by cytidine and uridine (Fig. 5C). This contrasts with other experimental systems, where cytidine is the most common and guanosine the least common (Legnini et al. 2019; Gumińska et al. 2025). Notably, gene-level analysis followed by closer inspection of Ninetails output and raw nanopore signals unveiled a subset of transcripts with tails significantly enriched in nonadenosines (Fig. 5D; Supplemental Figs. 6D,E, 7). The most notable example is the *Nrgn* gene, where nearly 60% of the tails were decorated with (often multiple) guanosines (Fig. 5E,F; Supplemental Fig. 7). According to APA analysis results, *Nrgn* has two PASs (PASs coordinates in BED format in Zenodo; Supplemental Tables 4, 5; Gumińska et al. 2024). Reads using the distal PAS have blank tails (i.e., containing exclusively adenosines), whereas reads using the proximal PAS have highly decorated tails with a strikingly repetitive nonadenosine distribution pattern (Fig. 5E,F). Further analysis revealed that this regularity is due to the semi-templated tail, as in this isoform the short genomically encoded 3′UTR has an extremely adenosine-rich 3′-end directly followed by poly(A) (Fig. 5E,F; Supplemental Fig. 7B).

Similar to the semi-templated variant of *Nrgn*, other potential mRNA isoforms with semi-templated tails also originate from highly A/T-rich 3′UTR, using either alternative or canonical PASs. In total, we identified 189 transcripts (e.g., *Bdnf*, *Camk2b*, *Homer1*, *Nrgn*, *Phactr1*, *Tmed9*) that possess semi-templated tails (Fig. 5F,G; Supplemental Table 5), suggesting that in the experimental system studied by us, the high prevalence of guanosine arises from sources other than TENT4A/B activity. Notably, the most enriched GO-terms linked to these transcripts are related to cytoskeletal reorganization, synapse structure, dendrite development, neuroplasticity, and learning (Supplemental Fig. 6E). These findings suggest a previously unrecognized layer of gene regulation mediated by semi-templated tailing.

### Polyadenylation in the synaptic compartment is not induced by NMDA-R stimulation in vitro

**FIGURE 6.**
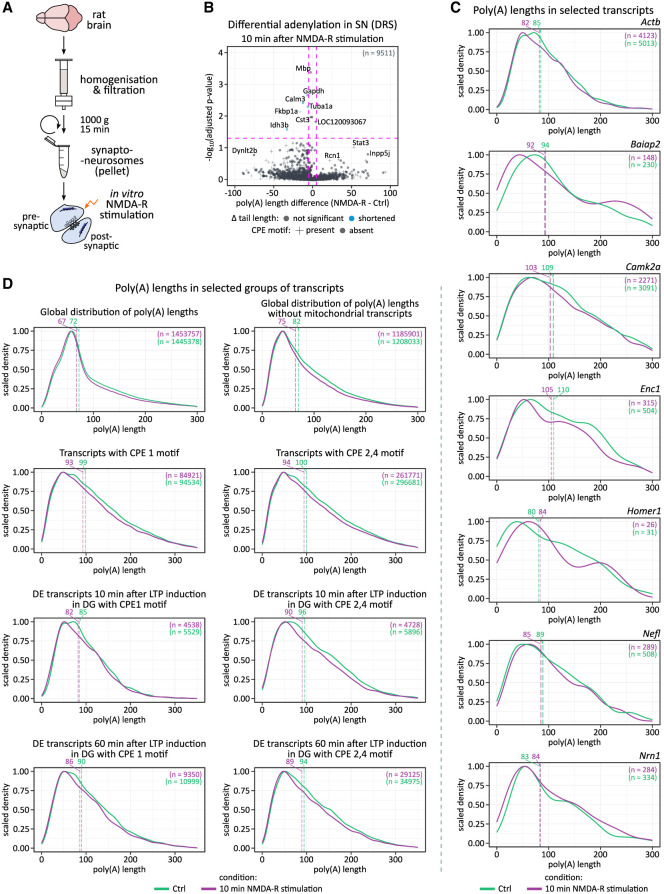
Poly(A) tail length distribution in synaptoneurosomes 10 min after in vitro NMDA-R stimulation (DRS). (*A*) Schematic depiction of synaptoneurosomes isolation and in vitro stimulation. (*B*) Polyadenylation profile of mRNAs in synaptoneurosomes upon NMDA-R stimulation. *P-*values between conditions were calculated using Wilcoxon signed-rank test, two-sided, α = 0.05, with Benjamini–Hochberg adjustment. The dashed lines divide the plot area into sectors based on *P*-value cut-off points and differences in poly(A) tail lengths. Zoomed view [cut-off −log_10_(adj. *P*-value) = 4] highlighting statistically significant gene alterations. Most mitochondrial genes were omitted for clarity (high adj. *P*-value, unchanged tail length). Unscaled version of this plot is provided in Supplemental Figure 8. (*C*) Poly(A) tail length distribution of selected transcripts involved in synaptic plasticity in synaptoneurosomes, measured by direct RNA sequencing (DRS). Dashed lines indicate median tail lengths for each condition, with median values shown above the plotting area. The total number of reads per condition (*n*) is also indicated. (*D*) Poly(A) tail length distribution of selected transcript groups in synaptoneurosomes, measured by direct RNA sequencing (DRS). Dashed lines indicate median tail lengths for each condition, with median values shown above the plotting area. The total number of reads per condition (*n*) is also indicated.

Potential cytoplasmic polyadenylation in our hippocampal data might have been obscured by highly expressed transcripts from nonneuronal cells or by the predominant effect of transcriptional induction (i.e., the influx of newly synthesized long-tailed molecules; see Fig. 2A) (Eisen et al. 2022). We therefore sought to identify transcripts undergoing local polyadenylation in the synaptic compartment, synaptoneurosomes stimulated chemically in vitro (Fig. 6A; for details, see Materials and Methods) (Kuzniewska et al. 2018, 2020). Enrichment and stimulation of synaptoneurosomes were performed using our optimized protocol, which effectively activates translation, leading to de novo protein synthesis (Kuzniewska et al. 2018, 2020). The effectiveness of protocol can be confirmed by western blotting (e.g., depletion/enrichment of specific markers and phosphorylation of ERK kinases; Supplemental Fig. 8A,B).

We performed DRS of mRNAs isolated from NMDA-R-stimulated synaptoneurosomes and corresponding controls to gain an accurate view of extranuclear polyadenylation (Fig. 6B). As expected, we observed only mild differences in transcript abundance between the conditions (Supplemental Figs. 8, 9A; Supplemental Table 6). The poly(A) profile was also largely unchanged (Fig. 6C; Supplemental Fig. 8C,D). Specifically, in stimulated synaptoneurosomes, no mRNAs displayed a significant shift in the poly(A) lengths from short toward longer tails that would be expected to occur if cytoplasmic polyadenylation played an important role in translational induction (Eisen et al. 2020, 2022). Instead, we observed a very small fraction of oligoadenylated mRNAs and a general slight shortening of poly(A) tail lengths occurring after the stimulation compared to controls. Analyzing poly(A) tail length distribution in transcripts related to synaptic plasticity also failed to confirm local cytoplasmic polyadenylation upon synaptoneurosomes stimulation (Fig. 6C,D; Supplemental Fig. 8D). There were also fewer nonadenosines in stimulated synaptoneurosomes than in corresponding controls (Supplemental Fig. 8E–G). Interestingly, mRNA isoforms with semi-templated tails exhibited a similar frequency in dentate gyrus and synaptoneurosomes: The semi-templated variant of *Nrgn*, for example, was only slightly reduced in synaptoneurosomes compared to dentate gyrus (Supplemental Fig. 8H). Moreover, analysis of *Nrgn* tail length distributions revealed comparable profiles across the two compartments, despite the overall trend toward poly(A) tail shortening in synaptoneurosomes versus dentate gyrus (Supplemental Fig. 9). Notably, stimulated synaptoneurosomes showed a modest shift toward longer tails in semi-templated variant of *Nrgn*. These observations indicate that semi-templated poly(A) tails may be relatively resistant to cytoplasmic shortening, which is consistent with the results of in vitro experiments with synthetic reporters (Lee et al. 2024).

**FIGURE 7.**
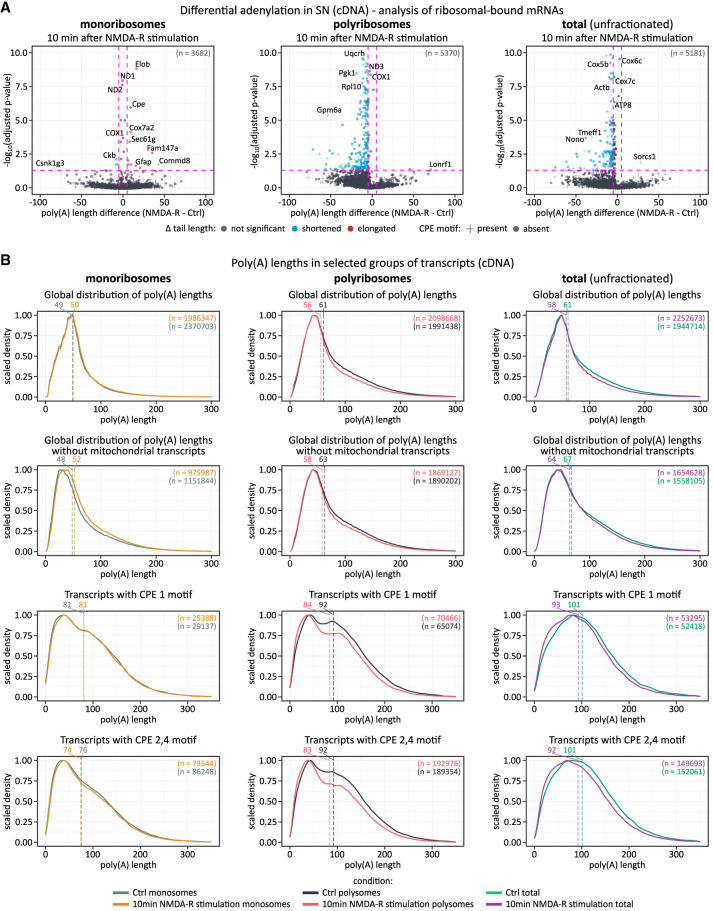
Poly(A) tail length distribution in synaptoneurosome-derived fractions containing mRNA bound to monosomes, polysomes, and total mRNA, 10 min after in vitro NMDA-R stimulation (cDNA sequencing). (*A*) Polyadenylation profile of ribosomal-bound mRNAs in synaptoneurosomes fractions upon NMDA-R stimulation. *P*-values between conditions were calculated using Wilcoxon signed-rank test, two-sided, α = 0.05, with Benjamini–Hochberg adjustment. Dashed lines divide the plot area into sectors based on *P*-value cut-off points and differences in poly(A) tail lengths. Zoomed view [cut-off −log_10_(adj. *P*-value) = 10] highlighting statistically significant gene alterations. Most mitochondrial genes were omitted for clarity (high adj. *P*-value, unchanged tail length). Unscaled versions of these plots are provided in Supplemental Figure 10. (*B*) Poly(A) tail length distribution in synaptoneurosome-derived fractions containing mRNA bound to monoribosomes, polyribosomes, and total (unfractionated) mRNA, based on cDNA sequencing of selected transcript groups. (*Left* to *right*) Monoribosomal, polyribosomal, and total mRNA data. Dashed lines indicate median tail lengths for each condition, with corresponding median values shown above the plot. The total number of reads per condition (*n*) is also indicated.

As a next step to better understand the relationship between poly(A) tail length and local translation in synaptoneurosomes, we performed nanopore cDNA sequencing of polyribosome-associated mRNAs. Monosomes and polysomes were separated on sucrose gradients using extracts from both NMDA-R–stimulated and control synaptoneurosomes. Notably, the mRNAs associated with monosomes have significantly shorter tails than those associated with polysomes (Fig. 7; Supplemental Figs. 10–12; Supplemental Table 6), indicating a positive relationship between poly(A) tail length and translation in synapses. However, for both monosomes and polysomes alike the total fraction, the stimulation, led to a (slight) global shortening of poly(A) tails (Fig. 7; Supplemental Figs. 10–12), suggesting increased translation-dependent deadenylation rather than cytoplasmic polyadenylation. It further corroborated with the indication of the existence of presumably arrested polysomes and monosomes in neurons (Darnell et al. 2011).

Differential adenylation analysis comparing stimulated and unstimulated fractions of polyribosomes did not reveal clear evidence for the presence of mRNAs that undergo cytoplasmic polyadenylation. NMDA-R stimulation did not lead to a systematic increase in poly(A) tail lengths of any mRNAs known to be involved in synaptic plasticity in either monosome or polyribosomal fractions (Fig. 7B; Supplemental Figs. 10–12). Instead, most transcripts possessed either stable or slightly shortened poly(A) tails following stimulation (Fig. 6E), indicating that local translation in synaptoneurosomes most likely does not depend on cytoplasmic polyadenylation as a key regulatory mechanism.

## DISCUSSION

Local protein synthesis at synapses is regulated by multiple post-transcriptional mechanisms, ensuring rapid, on-demand translation in response to neuronal activity. Given the spatial separation between the synapses and the nucleus, translational control plays a dominant role over transcriptional mRNA regulation. This is evident even in simplified in vitro systems, where isolated synaptic fractions exhibit robust protein synthesis activation upon stimulation (Kuzniewska et al. 2018, 2020). In our study, we complemented in vivo LTP in the rat hippocampus with in vitro stimulation of synaptoneurosomes and applied long-read sequencing technologies: direct RNA and cDNA sequencing. This approach enabled an unbiased view of poly(A) tail dynamics and mRNA 3′-end processing, offering comprehensive resources on APA site usage, tail length distribution, and its nucleotide composition. Several benchmarking studies have addressed the accuracy and reproducibility of nanopore technology in terms of yield and poly(A) tail measurements (Krause et al. 2019; Sessegolo et al. 2019; Workman et al. 2019; Tudek et al. 2021; Brouze et al. 2024; Czarnocka-Cieciura et al. 2024; Krawczyk et al. 2024; Gumińska et al. 2025). These findings advocate for using this platform in comparative analyses (Garalde et al. 2018; Krause et al. 2019; Workman et al. 2019; Bilska et al. 2020; Drexler et al. 2021; Gewartowska et al. 2021; Guegan et al. 2022; Liudkovska et al. 2022; Krawczyk et al. 2025).

Cytoplasmic polyadenylation has been proposed as a key mechanism controlling local translation at synapses (Huang et al. 2023). Initially characterized in developing oocytes and early embryos (Richter 2007), this process involves the activation of dormant mRNAs with short poly(A) tails, which are elongated by cytoplasmic poly(A) polymerases, thereby rapidly initiating protein synthesis. RNA-binding proteins, particularly the CPEB family, have been implicated as major regulators of this process. However, our findings challenge this model in hippocampal neurons.

Our dentate gyrus sampling 10 and 60 min post-stimulation designed to capture well-defined early and late phases of LTP (Otani and Abraham 1989; Messaoudi et al. 2002, 2007; Ryan et al. 2011; Udagawa et al. 2012; Panja et al. 2014; Maag et al. 2015; Patil et al. 2023) did not reveal stimulation-induced poly(A) tail elongation. While we cannot exclude the possibility of transient or spatially restricted polyadenylation events outside this window, a mechanism that plays a predominant regulatory role would be expected to elicit detectable changes at these stages. However, we also observed consistent shortening of the poly(A) tail in synaptoneurosomes upon NMDA-R stimulation instead of elongation. Even nanopore sequencing of ribosome-associated mRNAs revealed that NMDA-R stimulation did not induce cytoplasmic polyadenylation in translating transcripts. Instead, most poly(A) tails remained stable or exhibited slight shortening in both mono- and polyribosomal fractions. Given that synaptoneurosomes offer synchronized and stimulus-responsive synaptic compartments, any robust cytoplasmic polyadenylation should be amplified in this model.

Discussing the known targets of canonical CPEB polyadenylation, the data presented herein provide no evidence for cytoplasmic polyadenylation of Camk2a in all our experimental systems. Moreover, we did not observe stimulation-induced cytoplasmic polyadenylation in other transcripts with predicted canonical CPE motifs, although this set included numerous well-established CPEB targets, supporting the validity of our bioinformatic survey. Nevertheless, possibly, a more comprehensive identification of mRNAs regulated by CPEBs achieved using CLIP-Seq, RIP-Seq, or PAR-CLIP (Stepien et al. 2016; Duran-Arqué et al. 2022; Poetz et al. 2022) would be essential for the identification of rare mRNA species undergoing poly(A) tail elongation upon stimulation.

It also remains possible that cytoplasmic polyadenylation events require higher spatial or temporal resolution to observe, particularly during later consolidation phases or in region-specific contexts, as previously suggested for DG versus CA1 circuitry. Approaches such as in situ or metabolic labeling may be necessary to detect localized polyadenylation events masked in homogenized samples (Drexler et al. 2021; Ietswaart et al. 2024).

The data we provide also raise general questions about the physiological role of noncanonical poly(A) polymerases in hippocampal neurons. While the relatively abundant TENT2 (GLD2) has been proposed to mediate CPEB-regulated cytoplasmic polyadenylation (Udagawa et al. 2012; Mansur et al. 2016), the recent data do not support its widespread activity in synaptic compartments (Wardaszka-Pianka et al. 2025). Similarly, TENT4A and TENT4B (GLD4), known for incorporating guanosines into poly(A) tails (Lim et al. 2018), appear to play a minor role, as guanosine incorporation was rare except in semi-templated tails. Furthermore, members of another family of cytoplasmic poly(A) polymerases, TENT5, exhibit very low expression in the hippocampus, so their impact can be ignored.

Overall, our data indicate that poly(A) tail dynamics observed in hippocampal neurons following LTP is primarily driven by transcriptional induction rather than cytoplasmic polyadenylation. The shorter poly(A) tails observed in synaptoneurosomes likely reflect the absence of recently transcribed mRNAs and active translation of quiescent, preexisting mRNA. Instead of poly(A) tail elongation, the global activation of translation following NMDA-R stimulation correlates with mild poly(A) tail shortening, consistent with cotranslational deadenylation. This is further supported by the enrichment of shorter-tailed mRNAs in synaptoneurosomes and polyribosomal fractions post-stimulation. Together, these results argue against cytoplasmic polyadenylation as a major driver of local translation in hippocampal neurons. Given the growing evidence that deadenylation is a cotranslational event, the global activation of translation associated with the deadenylation process explains our observation (Bae and Coller 2022).

Additionally, our analysis of alternative polyadenylation revealed widespread shifts toward proximal polyadenylation sites during in vivo LTP, consistent with previous reports in brain slices (Fontes et al. 2017). This preference for shorter 3′UTRs during heightened neuronal activity may enhance mRNA stability or evade miRNA-mediated repression (Fontes et al. 2017; Wang et al. 2022).

Finally, by analyzing poly(A) tail nucleotide composition, we identified a subset of mRNAs with semi-templated poly(A) tails. Many of these encode key synaptic proteins, such as neurogranin (*Nrgn*) (Jones et al. 2018). Notably, the semi-templated isoform of *Nrgn* displayed relatively stable tail length across conditions, with minimal changes observed following stimulation in both the dentate gyrus and synaptoneurosomes. Since nonadenosine residues inhibit deadenylation, these transcripts may be degraded at a slower rate, potentially prolonging their cytoplasmic half-life and maintaining their availability for localized translation (Lee et al. 2024). Future research using synthetic reporters mimicking semi-templated tail features could provide mechanistic insights into their influence on mRNA stability and translation. Additionally, more localized approaches combining metabolic labeling with fractionation and sequencing may enable precise temporal and spatial mapping of poly(A) tail dynamics at subcellular compartments, potentially unlocking the regulatory potential of these modifications (Drexler et al. 2021; Ietswaart et al. 2024). Nevertheless, our results emphasize the complexity of mRNA regulation at synapses and suggest that translation at synaptic sites is largely uncoupled from cytoplasmic polyadenylation.

In summary, our data do not support a major role for cytoplasmic polyadenylation in LTP, but we acknowledge that subtle or localized effects may have been missed. Further studies with higher spatial and temporal resolution are needed to fully understand its potential contribution to synaptic plasticity.

## MATERIALS AND METHODS

### Animals

In this study, male Sprague Dawley rats of the following ages were used: (I) 5 months old (eight individuals, for the analysis of dentate gyri), 4 weeks old (26 individuals, for experiments involving synaptosome preparations). Sequenced samples/individuals are detailed in Supplemental Table 1.

The animals were housed in the laboratory animal facility under a 12 h light–dark cycle with ad libitum access to food and water. Experimental procedures were approved by the Norwegian National Research Ethics Committee in accordance with EU Directive 2010/63/EU, ARRIVE guidelines. All experiments were conducted by the Federation of Laboratory and Animal Science Associations (FELASA) C-certified researchers.

### Stereotaxic surgery and electrode positioning

Rats were anesthetized with urethane (1.5 mg/kg, i.p.) and placed in a stereotaxic frame (David Kopf Instruments) once unresponsive to toe pinch. A thermostatically controlled heating pad was placed under the animal to maintain a temperature of 37°C. The scalp was incised longitudinally and retracted with bulldog clamps. Two burr holes were drilled rostral to bregma for ground and reference screws. For stimulation, a craniotomy was performed over the left hemisphere (7.9 mm caudal, 4.2 mm lateral, 2.5 mm ventral to bregma), targeting the angular bundle of the medial perforant path. For recording, a craniotomy was made over the same hemisphere (3.9 mm caudal, 2.3 mm lateral, ∼2.5–3 mm ventral to bregma), targeting the hilar region of the dentate gyrus. The recording electrode depth was determined when test pulses triggered the maximum field excitatory postsynaptic potential (fEPSP) slope.

### In vivo electrophysiology

The stimulating electrode delivered test pulses every 30 sec unilaterally into the medial perforant path to evoke fEPSPs in the hilar region of the dentate gyrus, measured extracellularly using a recording electrode. Stimulus intensities (typically 100–500 µA) were set to elicit one-third of the maximum population spike. Baseline potentials were recorded for 24 min. Following baseline acquisition, the current was doubled, and high frequency stimulation (HFS) was applied to induce long-term potentiation (LTP). Simulation was delivered via a Grass S88 Dual output stimulator (Grass Medical Instruments) using a three-session paradigm (5 min intervals), totaling 10 min 30 sec. Each session comprised four trains of eight pulses at 400 Hz with 10 sec intertrain intervals resulting in 96 pulses (Panja et al. 2014). Post-HFS, the current was immediately reduced to baseline levels, and recordings continued at 0.033 Hz for either 10 or 60 min, depending on the experimental group.

fEPSPs were recorded using Data Wave Technologies Work Bench Software. The fEPSP slope was determined as the maximum positive *dV*/*dt* within a defined time window, from onset to one-third of the positive peak, capturing early EPSPs characteristic of the monosynaptic connection from the entorhinal cortex to the dentate gyrus. Slope and population spike amplitude data were processed in Microsoft Office Excel 2010 (Microsoft Corporation), averaged over 2 min bins (mean of four consecutive measurements), and normalized to the 24 min baseline mean. GraphPad Prism was used for statistical analysis, comparing baseline to post-HFS values.

### Brain dissection

Immediately after the experiment, the rat was removed from the stereotax. For dissection: the brain was removed from the skull and placed on filter paper above a glass plate cooled with ice. Ice-cold saline solution was used periodically through dissection to cool the brain down. With the hippocampus isolated, blood vessels and connective tissue were removed before the cornu ammonis regions were separated from the dentate gyrus, and each was put in cold microtubes. The samples were put on dry ice and kept at −80°C. This was done for both the ipsilateral and contralateral dentate gyri.

### RNA extraction and quality check

Tissue was maintained on dry ice prior to homogenization in 1 mL of TRIzol using 1 mL glass/glass Dounce homogenizer. The homogenate was transferred to 1.5 mL LoBind type Eppendorf tube and centrifuged at 12,000*g* for 5 min at 4°C to remove lipid content. RNA was then extracted from the supernatant following the manufacturer's protocol. Precipitation was performed by adding glycogen (GlycoBlue Coprecipitant, 15 mg/mL; 30 µg per sample), 1 volume of isopropanol, and incubating overnight at −20°C. The RNA pellet was dissolved in RNase-free ddH_2_O and initially assessed for quality using a NanoDrop 2000 spectrophotometer, yielding ∼20 µg per sample. Further purification was performed using KAPA Pure Beads (KAPA Biosystems) according to the manufacturer's instructions. RNA integrity number (RIN) was determined using an Agilent Bioanalyzer (Agilent RNA 6000 Nano Kit). For synaptoneurosomes, RNA was isolated using the same method, with ∼1 mL TRIzol per 100 mg pellet.

### qRT-PCR

One microgram of RNA samples were reverse transcribed using random primers (GeneON #S300; 200 ng/RT reaction) and SuperScript IV Reverse Transcriptase (Thermo Fisher Scientific). Next, the cDNA samples were amplified using specific primers:

- c-fos Fwd: 5′-CCCATCCTTACGGACTCCC-3′
- c-fos Rev: 5′-GAGATAGCTGCTCTACTTTGCC-3′
- Arc Fwd: 5′-AAGTGCCGAGCTGAGATGC-3′
- Arc Rev: 5′-CGACCTGTGCAACCCTTTC-3′
- Gapdh Fwd: 5′-ATCAAGAAGGTGGTGAAGCA-3′
- Gapdh Rev: 5′-CATACCAGGAAATGAGCTTC-3′).

Each reaction was set in a final volume of 15 μL, using PowerUp SYBR Green Master Mix (Thermo Fisher Scientific A25742) in a LightCycler 480 (Roche). Fold changes in expression were determined using the ΔΔ*Ct* (where *Ct* is the threshold cycle) relative quantification method. Values were normalized to the relative amounts of *Gapdh* mRNA.

### Nanopore direct RNA sequencing

The DRS was performed as described by Bilska et al. (2020). Briefly, for dentate gyri, 4.5–5 μg of total RNA was mixed with 250 ng of oligo(dT)-enriched RNA from *Saccharomyces cerevisiae* and 5 ng of in vitro transcribed poly(A) standards. For synaptoneurosomes, 3 μg of cap-enriched RNA was mixed with 200 ng of *S. cerevisiae* oligo(dT)-enriched RNA and 5 ng of poly(A) standards. Libraries were prepared using the Direct RNA Sequencing Kit SQK-RNA002 (ONT) according to the manufacturer's instructions. Sequencing used FLO-MIN106D (9.4.1) flow cells on a MinION device controlled by MinKNOW software (ONT). Basecalling was performed with Guppy (ONT).

### Nanopore cDNA sequencing

For dentate gyri, 200 ng of total RNA was used to prepare nanopore cDNA sequencing libraries with the cDNA-PCR Sequencing Kit SQK-PCS111 (ONT) according to the manufacturer's instructions. Sequencing was carried out using FLO-MIN106D (9.4.1) flow cells on a MinION device controlled by MinKNOW software (ONT). The basecalling was performed with Guppy (ONT) and Dorado (ONT).

For synaptoneurosomes fractionated with linear sucrose gradient, 500 ng of RNA isolates was used to prepare libraries with PCR-cDNA Barcoding Kit SQK-PCB111-24 (ONT) according to the manufacturer's instructions. Sequencing was performed using FLO-MIN106D (9.4.1) flow cell with the MinION device controlled by MinKNOW software. Obtained reads were basecalled and poly(A)-profiled using Dorado (ONT).

### Poly(A) tail length profiling

Basecalled RNA reads were mapped to mRatBN7.2-derived reference transcriptome using minimap2 (Li 2018) (-k 14 -ax map-ont ‐‐secondary = no) and processed using SAMtools (Danecek et al. 2021) to filter out supplementary alignments and reads mapping to the reverse strand (samtools view -b -F 2320). The lengths of the poly(A) tails were estimated using the Nanopolish-polya function (Workman et al. 2019). Only reads tagged by Nanopolish as PASS or SUFFCLIP were considered in the following analyses.

The lengths of poly(A) tails in cDNA reads were estimated during basecalling with Dorado (‐‐estimate-poly-a), extracted from the source files, and aggregated in tabular form.

The NanoTail package (Krawczyk et al. 2024) was used to perform statistical analysis. The *P*-values were calculated using Wilcoxon signed-rank test (two-sided, α = 0.05) for transcripts represented by 10 or more reads and adjusted for multiple comparisons with Benjamini–Hochberg method (α = 0.05). Transcripts were considered significantly altered in poly(A) tail length if the adjusted *P*-value was <0.05 and the difference in tail length was >5 nt between the hemispheres. Cohen's *d* was used as an effect size measure. For values *x* < 0.2, effect size was considered “negligible,” for values 0.2 ≤ *x* < 0.5, it was considered “small,” for values 0.5 ≤ *x* < 0.8, it was considered “medium,” and for values *x* ≥ 0.8, it was considered “large.”

### Differential expression

Reads were mapped to the mRatBN7.2 reference genome using minimap2 (Li 2018) (-ax splice ‐‐secondary = no -uf). The read distribution across genes was summarized using featureCounts from the Subread package (Liao et al. 2014) (-L ‐‐fracOverlap 0.5 ‐‐fracOverlapFeature 0.5 -s 1). Reads overlapping multiple features and multimappers were excluded. Differential expression analysis was performed using DESeq2 (Love et al. 2014) with default settings. For visualization clarity, log_2_ fold-change estimates were shrunken using *apeglm* (Zhu et al. 2019) method.

### Nucleotide composition of poly(A) tails

Nonadenosine nucleotides in DRS reads were assessed with Ninetails 1.0.3 (Gumińska et al. 2025) (check_tails function; pass_only = FALSE, qc = TRUE). The raw classification results were further processed and visualized using Ninetails’ postprocessing functions with default parameters.

The last 60 nt of each annotated 3′UTR were extracted from the mRatBN7.2 FASTA reference based on the GCF_015227675.2 reference annotation. Antisense strand sequences were reverse-complemented, and A/T percentages were calculated. Transcripts meeting at least one of the following criteria were retained: (I) A/T content >60% (147 transcripts) or (II) ≥13 consecutive A/T nucleotides, with the last three also being A/T (406 transcripts). The 13 nt threshold was based on the R9 pore pentamer reading context, covering 2.5 independent nucleotide contexts—sufficient for detecting meaningful signal differences. After merging and deduplication, 471 transcripts with potential semi-templated tails were identified.

A 60 nt upstream context was extracted for PAS sites predicted by LAPA and TAPAS. Applying the same filtering criteria resulted in 518 transcripts after deduplication. After merging reference-based (471) and PAS-predicted (518) sets, 988 transcripts remained. Of these, only those with ≥10 DRS reads and ≥3 poly(A) tails with nonadenosine nucleotides were retained, yielding 189 transcripts potentially possessing semi-templated tails.

Analysis was performed in R using GenomicRanges (Lawrence et al. 2013), IRanges (Lawrence et al. 2013), Biostrings (Pagès et al. 2024), stringr (Wickham 2023), dplyr (Wickham et al. 2022), and Ninetails (Gumińska et al. 2025).

### Alternative polyadenylation

The cDNA reads were preprocessed using pychopper (ONT). Reads fulfilling quality criteria and rescued fused reads were combined, then mapped to the mRatBN7.2 reference genome with Minimap2 (Li 2018) (-ax splice ‐‐secondary = no -uf -k 14). Alternative polyadenylation site analysis was performed with TAPAS (Arefeen et al. 2018) and LAPA (Çelik and Mortazavi 2022).

For the analysis using the TAPAS software, the mRatBN7.2 NCBI reference annotation (GCF_015227675.2-RS_2023_06) (https://www.ncbi.nlm.nih.gov/datasets/genome/GCF_015227675.2/) was converted into the refFlat format using the gtfToGenePred script from UCSC tools (http://hgdownload.soe.ucsc.edu/admin/exe/linux.x86_64/). Since TAPAS requires all gene identifiers (column 2) to begin with the “NM_” prefix (https://github.com/arefeen/TAPAS/issues/2), this modification was applied manually in R environment. Additionally, the columns were reordered according to the format specified by the software developers.

For the analysis using the LAPA software, the same NCBI reference was refined using scripts from AGAT (Dainat 2024). First, intron coordinates were incorporated into the annotation using the agat_sp_add_introns.pl script. The resulting file was then converted to GTF format using the agat_convert_sp_gff2gtf.pl script. Since the LAPA package requires UTR sequences to be labeled in a nonstandard format (“five_prime_utr” and “three_prime_utr”), the annotation was further modified in terminal (sed -e “s/<5UTR\>/five_prime_utr/g” -e “s/<3UTR\>/three_prime_utr/g” [input file name] > [output file name], where the placeholders in square brackets should be replaced with the actual file names). All resulting custom files are provided on Zenodo (Gumińska et al. 2024). The raw predictions of LAPA were further processed in Python according to the instructions provided by LAPA developers (https://colab.research.google.com/drive/1QzMxCRjCk3i5_MuHzjozSRWMaJgdEdSI?usp=sharing).

Differential usage of poly(A) sites was calculated using Fisher's exact test (two-sided, α = 0.05). *P*-values were adjusted using the Benjamini–Hochberg method. Given PAS was considered as differentially expressed if adjusted *P*-value was ≤0.1.

### CPE motif search

Established CPE1 and CPE2,4 motif sequences represented as position-dependent letter-probability matrices (Duran-Arqué et al. 2022) were used to identify CPE motifs, recognized by CPEB1 and CPEB2-4, respectively. Motif searches were conducted separately using the FIMO software from the MEME suite (Grant et al. 2011) (parameters: ‐‐bgfile ‐‐nrdb‐‐ ‐‐thresh 1.0E-4).

### Synaptoneurosomes isolation and in vitro stimulation

Synaptoneurosomes were prepared as described previously (Kuzniewska et al. 2018). Briefly, Krebs buffer (2.5 mM CaCl_2_, 1.18 mM KH_2_PO_4_, 118.5 mM NaCl, 24.9 mM NaHCO_3_, 1.18 mM MgSO_4_, 3.8 mM MgCl_2_, and 212.7 mM glucose) was aerated at 4°C for 30 min using an aquarium pump, then adjusted to pH 7.4 with dry ice. The buffer was supplemented with 1× protease inhibitor cocktail (EDTA-free, Roche) and RNase Inhibitor (RiboLock, 60 U/mL, Thermo Fisher Scientific).

Rats were euthanized by cervical dislocation, and hippocampi were dissected. Tissue from one hemisphere was homogenized in a 1.5 mL of Krebs buffer using a Dounce homogenizer (10–12 strokes). To prevent the stimulation of synaptoneurosomes, all steps were performed on ice. Homogenates were passed gravitationally through presoaked nylon mesh filters (100, 60, 30, and 10 μm, Merck Millipore) into a 50 mL polypropylene tube, centrifuged at 1000*g* for 15 min at 4°C, washed, and resuspended in Krebs buffer with protease and RNase inhibitors.

In vitro stimulation of NMDA receptors on synaptoneurosomes was performed as described before (Kuzniewska et al. 2018). Briefly, freshly isolated synaptoneurosomes were prewarmed at 37°C for 5 min, stimulated with 50 μM NMDA and 10 μM glutamate for 30 sec, followed by 120 μM APV and incubation at 37°C for 10 min. Unstimulated samples kept on ice served as controls. Synaptoneurosomes from five rats were pooled and split into control and stimulated samples to produce enough material for Nanopore Direct RNA Sequencing (DRS). Four independent isolations were performed for DRS.

### Polysome profiling

Linear sucrose gradients (10%–50%) were prepared in GB buffer (10 mM HEPES–KOH pH 7.2, 150 mM KCl, 5 mM MgCl_2_, Protease Inhibitor Cocktail [Roche], 100 μg/mL cycloheximide [CHX; Thermo Fisher Scientific], 4 U/mL RiboLock [Thermo Fisher Scientific], and nuclease-free water). Krebs buffer was prepared as described in “Synaptoneurosomes isolation and in vitro stimulation” section.

Rats were sacrificed by cervical dislocation. Brains were rapidly harvested, briefly chilled in ice-cold Krebs buffer, and hippocampi were dissected and halved transversely. Tissue from one half was homogenized in 1.5 mL Krebs buffer using glass-glass Dounce homogenizer (10–12 strokes), and homogenates were pooled. All steps were performed on ice to prevent synaptoneurosome stimulation. Homogenates were further processed as described in the “Synaptoneurosomes isolation and in vitro stimulation” section, and resuspended in 1 mL of Krebs buffer with protease and RNase inhibitors.

Cycloheximide (100 µg/mL) was added to control synaptoneurosomes kept on ice. The second aliquot was prewarmed at 37°C (800 rpm, thermoblock) for 5 min, stimulated with 50 μM NMDA and 10 μM glutamate for 30 sec, followed by 120 μM of APV, and incubation for 10 min at 37°C (800 rpm, thermoblock). Stimulation was halted by adding cycloheximide (100 µg/mL) and incubating on ice for 2 min. Samples were centrifuged at 1000*g* for 5 min at 4°C, and synaptoneurosomes were resuspended in GB buffer with 1.5% NP-40. After 10 min on ice, samples were centrifuged at 20,000*g* for 15 min. RNA was extracted by mixing 300 μL of lysate with 900 μL of Tri reagent LS.

Equal volumes of samples (1.1 mL) were loaded onto sucrose gradients, and ultracentrifuged at 38,000 rpm for 2 h at 4°C using an Optima XPN ultracentrifuge with an SW41Ti rotor (Beckman Coulter). Sucrose fractions were collected using a Density Gradient Fractionation System (Teledyne ISCO) with a Foxy Jr. Fraction Collector. Fractions were stored at −80°C for further analysis. Absorbance at 254 nm was recorded during collection to generate RNA distribution profiles.

### Western blotting analysis of synaptoneurosomal preparations

Equal amounts of protein from homogenate and synaptoneurosomal fraction were resolved on SDS-PAGE (10%, TGX Stain-Free FastCast Acrylamide Solutions, Bio-Rad). After electrophoresis, proteins in the gel were visualized using Bio-Rad's ImageLab software to verify the equal protein loading. Proteins were transferred to PVDF membranes (pore size 0.45 µm, Immobilon-P, Merck Millipore) using the Trans-Blot Turbo Blotting System (Bio-Rad). Membranes were blocked for 1 h at room temperature in 5% nonfat dry milk in PBS-T (PBS with 0.01% Tween-20), followed by overnight incubation at 4°C with primary antibodies (catalog numbers and producers of antibodies are listed in Supplemental Table 1) in 5% milk in PBS-T. Blots were washed 3 × 5 min with PBS-T, incubated 1 h at room temperature with HRP-conjugated secondary antibody (1:10,000 in 5% milk), and washed 3 × 5 min with PBS-T. In the case of MAPK/Phospho-MAPK Family Antibody Sampler Kit, membranes were blocked for 1 h at room temperature in 5% bovine serum albumin (BSA) in PBS-T; primary and secondary antibodies were also diluted in 5% BSA in PBS-T. HRP signal was detected using Amersham ECL Prime Western Blotting Detection Reagent (GE Healthcare) on ChemiDoc Imaging System (Bio-Rad) using automatic detection settings.

### Statistics and reproducibility

Sample size was not predetermined using a statistical method. Statistical analyses were conducted on data from two or more biologically independent replicates. Quantitative data were analyzed within the R environment, with specific statistical tests detailed in the figure legends. Normality was assessed using the Shapiro–Wilk test. Most experiments were performed at least twice, producing consistent results.

### Data visualization

The visualization of the data was performed in the R environment. Poly(A) nucleotide composition analyses using Ninetails (Czarnocka-Cieciura et al. 2025; Gumińska et al. 2025). Heat maps were created with ComplexHeatmap (Gu 2022), and genome browser-like diagrams were drawn using ggcoverage (Song and Wang 2023). Gene ontology enrichment was performed using g:Profiler (Raudvere et al. 2019) and ClusterProfiler (Wu et al. 2021). Correlograms of gene expression profiles were drawn with corrplot (Wei and Simko 2024). The remaining diagrams were created using ggplot2 (Wickham 2016).

## DATA DEPOSITION

Raw sequencing data (fast5 files) are deposited at the European Nucleotide Archive (ENA) (https://www.ebi.ac.uk/ena/browser/home) under project accession numbers PRJEB75352 and PRJEB75356 with sample numbers listed in Supplemental Table 1. Source data used for plotting (including statistics) are provided in Supplemental Tables 2–6. Supplemental Tables and other resources (e.g., custom references, CPE motifs, APA site coordinates) are deposited in Zenodo (Gumińska et al. 2024) (https://doi.org/10.5281/zenodo.11478468). Supplemental Figures are provided in the Supplemental Material file. Additional files/code snippets and information are available from the authors upon request. Code availability: Custom software (NanoTail) used for poly(A) tail analysis in R is available on the GitHub repository (https://github.com/LRB-IIMCB/nanotail). Custom software (Ninetails) used for nonadenosine profiling in R is available on the GitHub repository (https://github.com/LRB-IIMCB/ninetails) and in the Zenodo repository (https://doi.org/10.5281/zenodo.11478467). Other programs, tools, and scripts used in this work are listed in Supplemental Table 1.

## SUPPLEMENTAL MATERIAL

Supplemental material is available for this article.

## Figures and Tables

**Natalia Gumińska:**
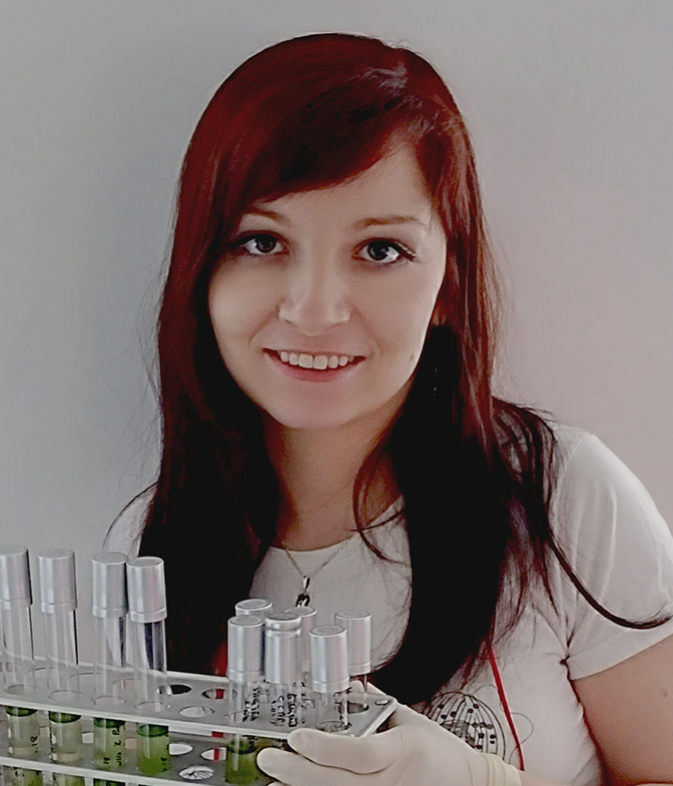


**Francois P. Pauzin:**
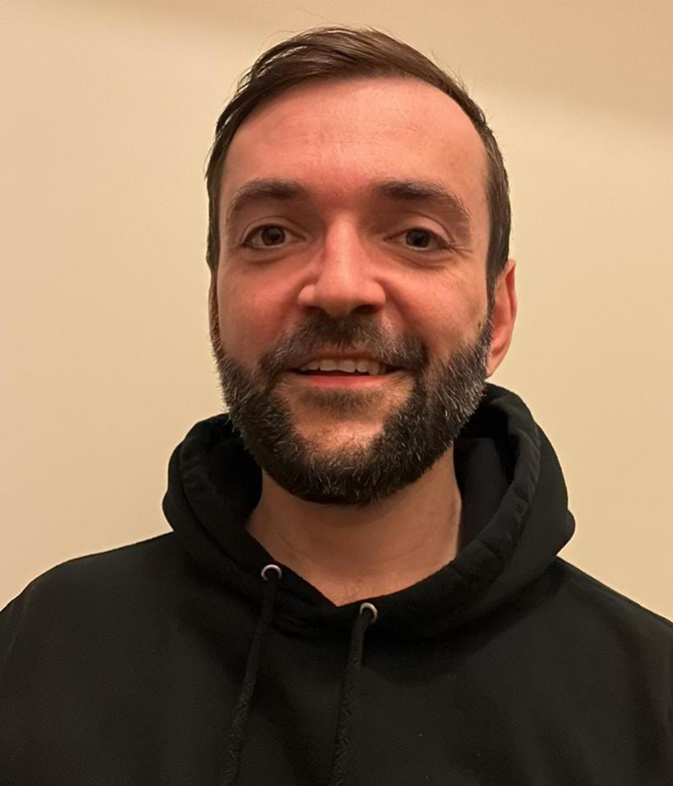


## MEET THE FIRST AUTHORS

***Meet the First Author(s)* is an editorial feature within *RNA*, in which the first author(s) of research-based papers in each issue have the opportunity to introduce themselves and their work to readers of *RNA* and the RNA research community. Natalia Gumińska and Francois Philippe Pauzin are co-first authors of this paper, “Polyadenylation landscape of in vivo long-term potentiation in the rat brain.” Natalia is a postdoc at the Laboratory of RNA Biology, International Institute of Molecular and Cell Biology in Warsaw, Poland. She is a bioinformatician particularly interested in poly(A) profiling and mixed tailing. Francois is a researcher at the Kavli Institute for Systems Neuroscience, Trondheim, Norway. He is an electrophysiologist particularly interested in sensory processing.**

**What are the major results described in your paper, and how do they impact this branch of the field?**

**NG:** Our study reveals that local cytoplasmic polyadenylation is not the major mechanism shaping the poly(A) tail landscape during LTP in hippocampal neurons. Even in synaptoneurosomes, where local effects should be most apparent, we did not observe evidence indicating local cytoplasmic polyadenylation as a global mechanism regulating poly(A) tail lengths. Furthermore, translating transcripts do not undergo cytoplasmic polyadenylation, and our data do not support widespread activity of TENT2 (GLD2) in synaptic compartments. These findings challenge the long-standing view that cytoplasmic polyadenylation is a major driver of local translation during synaptic plasticity. Additionally, we identified a pool of transcripts with mixed, semi-templated poly(A) tails, which raises further questions regarding poly(A) regulation.

**FPP:** This study is both a proof of method showing the robustness of the Nanopore sequencing in brain tissue and a study providing insight into mRNA 3′-end dynamics following synaptic plasticity. To our knowledge, we are the first to demonstrate that cytoplasmic polyadenylation is not a key regulator mechanism in long-term potentiation.

**What led you to study RNA or this aspect of RNA science?**

**NG:** Direct RNA sequencing from Oxford Nanopore is the only method that allows analysis of RNA in its native form, without relying on synthetic proxies. I find this a huge advantage over other methods, as it preserves information on RNA modifications and doesn't require additional chemical processing of the RNA sample, which I think is amazing. Poly(A) tails are particularly interesting to me because they play a crucial role in determining the fate of mRNA molecules within the cell, which sparked my curiosity. In the nervous system, the regulation of poly(A) tails is highly specific and influenced by multiple *cis*- and *trans*-acting elements. The deeper I explored this, the more fascinating it became, especially considering its implications for brain function and memory formation.

**FPP:** As an electrophysiologist, recording the dentate gyrus field potential and seeing clear LTP post-electrical stimulation is fascinating. It was even more captivating to scrutinize the basis of this potentiation by studying the regulatory mechanisms of local protein synthesis with a focus on cytoplasmic polyadenylation.

**During the course of these experiments, were there any surprising results or particular difficulties that altered your thinking and subsequent focus?**

**NG:** The most surprising finding was that local cytoplasmic polyadenylation was not the major player regulating mRNA poly(A) processing during long-term potentiation. We initially aimed to confirm previous results obtained with low-throughput methods and to identify more transcripts regulated by this mechanism. To our surprise, the data did not support local cytoplasmic polyadenylation as a key factor. Because this was unexpected, we carefully cross-validated our findings using synaptoneurosomes from brain slices. I believe our results could spark interesting discussion in the field.

**FPP:** To our knowledge, we are the first to demonstrate that cytoplasmic polyadenylation is not a key regulator mechanism in long-term potentiation. This is a result that indeed changed my way of seeing poly(A) tail length regulation.

**What are some of the landmark moments that provoked your interest in science or your development as a scientist?**

**FPP:** I guess that my curiosity toward understanding our surrounding world has pushed me toward being a doctor in philosophy. The influence of my mum as a mathematical teacher has undoubtedly led me toward science.

**If you were able to give one piece of advice to your younger self, what would that be?**

**FPP:** I would start writing grants during my master's studies. You have nothing to lose, go for it!

**Are there specific individuals or groups who have influenced your philosophy or approach to science?**

**NG:** It has grown from everything I've experienced: the people I've met, my family and friends, teachers, lecturers, the books and research papers I've read, and every bit of information my mind has processed along the way.

**FPP:** Meeting great scientists is undoubtedly the best way to be influenced. Thank you, Professor Patrik Krieger, Professor Karen Moxon, Professor Clive Bramham. And of course, thank you Natalia Gumińska.

**What are your subsequent near- or long-term career plans?**

**NG:** I plan to continue working on nanopore data, improving my current software and pipelines, and developing new ones. I aim to become more proficient in programming and then decide whether I want to pursue a position either as a principal investigator or find my place elsewhere.

**FPP:** I have always been a curious scientist with specific questions toward sensory processing in the cerebral cortex and the thalamus. I am aiming to open my own laboratory toward that direction.

**What were the strongest aspects of your collaboration as co-first authors?**

**NG:** The high degree of creative freedom we had, combined with the possibility to always consult with our PIs anytime we wanted to discuss the results and ideas.

**FPP:** The strongest aspect of our collaboration was the distance! Indeed, a group in Poland and a group in Norway joined forces to achieve this study.
